# Coronaviruses: emerging and re-emerging pathogens in humans and animals

**DOI:** 10.1186/s12985-015-0432-z

**Published:** 2015-12-22

**Authors:** Susanna K. P. Lau, Jasper F. W. Chan

**Affiliations:** State Key Laboratory of Emerging Infectious Diseases, The University of Hong Kong, University Pathology Building, Queen Mary Hospital, Hong Kong, China; Department of Microbiology, The University of Hong Kong, Hong Kong, China; Research Centre of Infection and Immunology, The University of Hong Kong, Hong Kong, China; Carol Yu Centre for Infection, The University of Hong Kong, Hong Kong, China

## Abstract

The severe acute respiratory syndrome coronavirus (SARS-CoV) and recently emerged Middle East respiratory syndrome coronavirus (MERS-CoV) epidemics have proven the ability of coronaviruses to cross species barrier and emerge rapidly in humans. Other coronaviruses such as porcine epidemic diarrhea virus (PEDV) are also known to cause major disease epidemics in animals wiith huge economic loss. This special issue in Virology Journal aims to highlight the advances and key discoveries in the animal origin, viral evolution, epidemiology, diagnostics and pathogenesis of the emerging and re-emerging coronaviruses in both humans and animals.

## Editorial

In the coming months, Virology Journal will publish a thematic series of reviews and original articles on emerging and re-emerging coronaviruses.

Several coronaviruses have caused serious problems in humans and animals in the past two decades. The best known examples are severe acute respiratory syndrome coronavirus (SARS-CoV), Middle East respiratory syndrome coronavirus (MERS-CoV) and porcine epidemic diarrhea virus (PEDV). Urbanization and the increasingly frequent mixing of different animals in densely populated areas may have facilitated the emergence and re-emergence of some of these viruses. On the other hand, coronaviruses are known to have high mutation and recombination rates, which may allow them to cross species barriers and adapt to new hosts.

The SARS epidemic in 2003 has awakened scientists and the world on the ability of coronaviruses for animal-to-human transmission. It was one of the worst epidemics in our city’s history, with 1,755 people infected and 299 died from the dreadful infection. The animal sources of SARS-CoV was later traced back to civets in wild life markets in China as the intermediate host, and ultimately to horseshoe bats as the primary reservoir [1, 2]. Although SARS-related coronaviruses are continuously found in bats from China and worldwide, it is believed that the immediate ancestor of human SARS-CoV has disappeared. Nevertheless, we still have to be vigilant about the possibility of re-emergence of SARS and uphold measures to avoid mixing of wild animals especially bats and civets in markets.

The SARS epidemic has boosted interests in hunting for other novel coronaviruses. Before the SARS epidemic, only two coronaviruses were known to infect humans, namely human coronavirus (HCoV)-229E and HCoV-OC43. Two additional human coronaviruses, HCoV-NL63 and HCoV-HKU1, were discovered soon after the SARS epidemic, although these viruses have likely been circulating in humans for a long time prior to their discovery. On 23 September 2012, the World Health Organization (WHO) announced the discovery of a new coronavirus, MERS-CoV, in two patients who died from a mysterious, rapidly-fatal disease in the Middle East [3]. The virus was, at that time, found to be most closely related to the prototype lineage C betacoronaviruses, *Tylonycteris* bat coronavirus HKU4 and *Pipistrellus* bat coronavirus HKU5, previously identified in Hong Kong [3–5]. Subsequently, dromedary camels were identified as the source of some human cases [6].

Apparently, MERS has an even higher mortality rate (>35 %) than SARS (9.6 %), which may be partly explained by the high prevalence of medical comorbidities among infected patients [7]. Fortunately, in the past three years, MERS has not been transmitted from person to person as efficiently as SARS was. However, a number of large healthcare-associated outbreaks in the Middle East and the recent epidemic in the Republic of Korea have highlighted the possibility of sustained person-to-person transmission of MERS-CoV. In the recent outbreak in Korea, more than 180 people were infected as a result of second-, third-, or fourth generation infection from a single index case who acquired MERS after traveling to Qatar. Although these large-scale outbreaks of MERS were eventually controlled with enhanced infection control measures, they have highlighted the importance of rapid diagnosis and coordinated public health response. A lot more research efforts on MERS-CoV are needed, especially at the epicenter, the Middle East, where continuous animal-to-human transmissions of MERS-CoV are still being regularly reported. This is in contrast to SARS which was rapidly contained after removal of civets from wild life markets in southern China. In particular, more research on the pathogenesis, treatment and prevention of this new coronavirus with pandemic potential are urgently needed [7].

Besides humans, coronaviruses also cause various diseases in animals with some resulting in significant economic losses. PEDV, a member of the genus *Alphacoronavirus*, is the etiological agent of porcine epidemic diarrhea in swine populations. The disease was endemic in Asia and European countries in the past decades. In 2013, the virus has emerged or re-emerged to cause large-scale epidemics in the Americas and Asia, causing millions of fatal cases among piglets and representing one of the most serious concerns among the global pig industries [8, 9]. The ability of epidemic PEDV strains to emerge despite current vaccination schemes suggests that the virus is able to evolve and escape from immune protection.

The special issue to be published in Virology Journal will emphasize advances and key discoveries in the animal origin, viral evolution, epidemiology, diagnostics and pathogenesis of different emerging and re-emerging coronaviruses.

Two reviews in this thematic series will focus on the evolution and pathogenesis of MERS-CoV. While both SARS and MERS can cause similar severe respiratory illnesses, considerable differences exist in terms of patient demographics, clinical outcomes as well as transmission dynamics. The review, *MERS coronavirus: epidemiology, transmission and diagnostics* by I. Mackay *et al.* discusses the current understandings on the epidemiology and transmission of MERS, and highlights the keys to rapid diagnosis [10]. While intensive research has been conducted on this virus, histopathological and immunopathogenesis studies using clinical samples are scarce and autopsy findings are lacking, partly due to cultural reasons. As a result, current understandings on the pathogenesis and immune response of MERS rely mainly on *in vitro* or *in vivo* studies. The review by J. Zhou *et al.* (*MERS coronavirus infection in human cells: virus-host interaction and implication for pathogenesis*) summarizes the most up-to-date findings on virus-host interaction of MERS-CoV based on cellular and animal studies and their implications on disease pathogenesis [11].

Bats are important reservoir for many emerging viruses. Similar to SARS-CoV, the finding of MERS-CoV-related bat coronaviruses suggests that bats may also be the ultimate animal origin of MERS-CoV, with camels being the intermediate hosts before the virus crossed species barriers to infect humans [7]. It is now known that bats are important reservoirs of diverse alphacoronaviruses and lineage B, C and D betacoronaviruses [12]. While horseshoe bats in China are the natural reservoir of SARS-CoV-like viruses, the bat species that may harbor the ultimate origin of MERS-CoV is yet to be identified. In a review entitled *Bat origin of coronaviruses*, Z. Shi *et al.* summarize the role of bats as the origin of emerging coronaviruses in humans, with special focus on SARS-CoV and MERS-CoV [13]. The review will also emphasize the importance of field studies to elucidate the animal origins of coronaviruses in predicting and preventing future pandemics.

Beside SARS-CoV and MERS-CoV which have only emerged recently, the other four human-pathogenic coronaviruses, namely, HCoV-229E, HCoV-OC43, HCoV-NL63 and HCoV-HKU1, have been continuously circulating in human for at least decades or centuries. Although they are often thought to be associated with mild respiratory illnesses, increasing reports have suggested that they may cause severe infections, especially in people at the extremes of age or those with comorbidities. However, pathogenesis studies are often limited by the difficulties in isolating these coronaviruses in cell cultures. The human airway serves as the first entry point for these respiratory viruses. We also plan to include a review that discusses the establishment of human airway epithelial cells for isolation of human coronaviruses and latest understandings on virus-host interactions based on cell culture studies.

Apart from humans, animals are also susceptible to different coronaviruses and PEDV represents a notable example how coronaviruses can cause serious epidemics in animal population worldwide. In the review entitled *Emergence and re-emergence of PEDV*, C. Lee *et al.* discuss the latest advances in understanding the epidemiology, pathogenesis and control measures of PEDV, focusing especially on the recent epidemics affecting America and Asia [14].

Virology Journal is taking a leading role in facilitating the dissemination of new information in various emerging infectious diseases including coronaviruses. It is hoped that the present series will stimulate more research and collaborative efforts to control the ongoing and prevent future epidemics caused by coronaviruses. We are grateful to Linfa Wang, Ph.D., Editor in Chief of Virology Journal and Professor at Duke-National University of Singapore for his guidance during the preparation of this thematic series.
